# *In Vivo* Imaging of Influenza Virus Infection in Immunized Mice

**DOI:** 10.1128/mBio.00714-17

**Published:** 2017-05-30

**Authors:** Rita Czakó, Leatrice Vogel, Elaine W. Lamirande, Kevin W. Bock, Ian N. Moore, Ali H. Ellebedy, Rafi Ahmed, Andrew Mehle, Kanta Subbarao

**Affiliations:** a Laboratory of Infectious Diseases, NIAID, NIH, Bethesda, Maryland, USA; b Comparative Medicine Branch, Infectious Disease Pathogenesis Section, NIAID, NIH, Bethesda, Maryland, USA; c Emory Vaccine Center, Emory University, Atlanta, Georgia, USA; d Department of Medical Microbiology and Immunology, University of Wisconsin at Madison, Madison, Wisconsin, USA; Virginia Polytechnic Institute and State University

## Abstract

Immunization is the cornerstone of seasonal influenza control and represents an important component of pandemic preparedness strategies. Using a bioluminescent reporter virus, we demonstrate the application of noninvasive *in vivo* imaging system (IVIS) technology to evaluate the preclinical efficacy of candidate vaccines and immunotherapy in a mouse model of influenza. Sequential imaging revealed distinct spatiotemporal kinetics of bioluminescence in groups of mice passively or actively immunized by various strategies that accelerated the clearance of the challenge virus at different rates and by distinct mechanisms. Imaging findings were consistent with conclusions derived from virus titers in the lungs and, notably, were more informative than conventional efficacy endpoints in some cases. Our findings demonstrate the reliability of IVIS as a qualitative approach to support preclinical evaluation of candidate medical countermeasures for influenza in mice.

## IMPORTANCE

Influenza A viruses remain a persistent threat to public health. Vaccination and immunotherapy are effective countermeasures for the control of influenza but must contend with antigenic drift and the risk of resistance to antivirals. Traditional preclinical efficacy studies for novel vaccine and pharmaceutical candidates can be time-consuming and expensive and are inherently limited in scope. *In vivo* imaging approaches offer the potential to noninvasively track virus replication in real time in animal models. In this study, we demonstrate the utility of bioluminescent imaging for tracking influenza virus replication in the lungs of immunized mice and also identify important factors that may influence the accurate interpretation of imaging results. Our findings support the potential of IVIS approaches to enhance traditional preclinical efficacy evaluation of candidate vaccines and human monoclonal antibodies for the prevention and treatment of influenza.

## INTRODUCTION

Seasonal circulation of influenza viruses affects an estimated 5 to 15% of the global population (1) and is responsible for over 24,000 excess deaths per year in the United States alone (2). Sporadic outbreaks associated with zoonotic introduction of novel viruses serve as a reminder of the potential for another influenza pandemic. As public health experience from the 2009 H1N1 pandemic has illustrated, early intervention with effective countermeasures is critically important yet fraught with complications in practice (3). In the absence of a universal influenza vaccine or immunotherapy, the development and evaluation of vaccines, novel antiviral pharmaceuticals, and monoclonal antibodies (MAbs) remain a priority.

Candidate vaccines and antiviral agents for influenza virus are routinely evaluated in mice and ferrets, but these studies can be time-consuming and require large numbers of animals. *In vivo* imaging system (IVIS) technology offers great promise as an alternative or adjunct to traditional preclinical efficacy studies. Recent advances in imaging technology and reporter enzyme biochemistry have made the use of IVIS feasible in small mammals (4). Because it is noninvasive, IVIS permits repeated assessment of viral infection in the same cohort of animals and thus reduces the minimum number of animals necessary per intervention group without compromising statistical power. Imaging also offers theoretical advantages in the context of sublethal challenge, heterosubtypic immunity, or therapeutic intervention, where the spatiodynamics of viral replication may be more informative than the traditional endpoints of survival or virus titration at predetermined time points.

Proof of concept for *in vivo* imaging of influenza virus infection has been demonstrated in immunologically naive animals (5–7) and in limited assessment of relative antiviral or antibody efficacy at a single time point (8, 9). However, the reliability of IVIS as a tool for real-time tracking of viral loads in the context of a preclinical efficacy study and the extent to which preexisting immunity may influence imaging findings are not known. Several strategies for the production of fully infectious, pathogenic, and genetically stable reporter influenza A viruses have been described (6–12). For our studies, we selected the strategy described by Tran et al., based on its genetic stability, near-native properties of virulence and pathogenicity in mice, and incorporation of the small yet very bright engineered luciferase NanoLuc (NLuc) (8).

We report that noninvasive imaging can identify distinct patterns of bioluminescence associated with protection mediated by different immunization strategies. Specifically, mice vaccinated with homologous versus heterosubtypic live attenuated influenza vaccines can readily be distinguished on the basis of bioluminescence kinetics. Imaging can also detect different patterns of virus replication in mice passively immunized with antibodies directed to the stem versus the head domain of the influenza virus hemagglutinin (HA), and changes in bioluminescence in some cases preceded changes in weight loss and/or virus titers. Finally, we also describe factors that may influence the reliability of imaging results. Overall, these findings establish that IVIS is a promising platform for preclinical evaluation of candidate vaccines and antibodies for influenza virus and suggest that it has the potential to enhance mechanistic studies of protection associated with distinct immunization strategies.

## RESULTS

### Replication of H1N1pdm09-NLuc *in vitro*.

**FIG 1:**
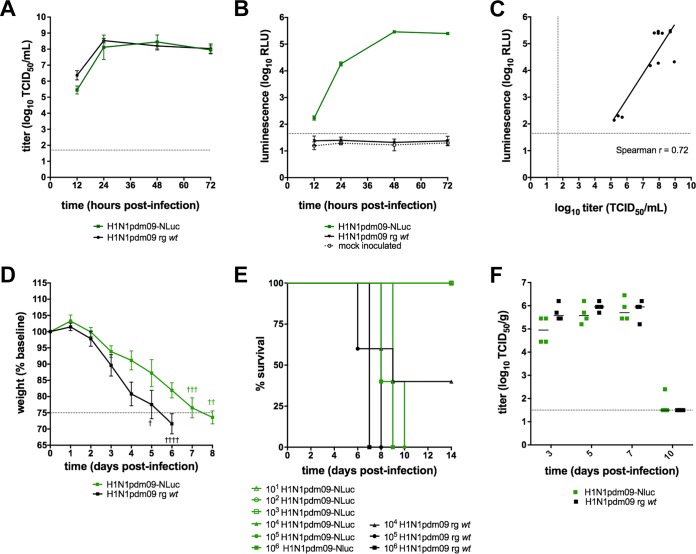
H1N1pdm09-NLuc is not attenuated *in vitro* or in BALB/c mice. MDCK cells were inoculated in triplicate at an MOI of 0.01 with either RG *wt* H1N1pmd09 or RG H1N1pdm09-NLuc. Supernatant was collected at the time points indicated for virus titration (A) or quantification of the luminescent signal (RLU, relative light units) (B). The correlation between supernatant luminescence and infectious virus titers (C) of H1N1pdm09-NLuc were estimated by Spearman’s rank order correlation (*r* = 0.72 [95% CI, 0.23 to 0.92]; *P* = 0.01). (D, E) Weight loss (mean ± standard deviation) (D) and survival (E) were analyzed in BALB/c mice (five per group) inoculated with either H1N1pdm09-NLuc or RG *wt* H1N1pdm09 virus. (F) In a separate experiment, mice (four per group) were challenged with 10^3^ TCID_50_ of either H1N1pdm09-NLuc or RG *wt* virus. Infectious virus titers in lung homogenates were determined at the time points indicated. Dashed lines indicate the limits of detection of infectious virus (A, C, F) and the bioluminescent signal (B, C) or the threshold for euthanasia due to weight loss associated with infection (D).

A bioluminescent reporter virus (H1N1pdm09-NLuc) was generated by reverse genetics (RG) to evaluate the performance of *in vivo* imaging data as a “biomarker” of virus replication in preclinical efficacy studies for influenza vaccines and immunotherapeutics in mice. To confirm that H1N1pdm09-NLuc was fully infectious, we verified that the replication kinetics of the reporter virus were comparable to those of the RG wild type (*wt*) H1N1pdm09 virus *in vitro* (Fig. 1A). Supernatant was assayed to detect the luminescent signal; luminescence kinetics correlated with the accumulation of infectious virus in the culture supernatants (Fig. 1B and C).

### H1N1pdm09 is infectious and pathogenic in mice.

To confirm that the reporter virus is fully infectious and pathogenic *in vivo*, we determined the median lethal dose of the bioluminescent reporter virus in adult BALB/c mice. Mice inoculated with H1N1pdm09-NLuc presented clinical signs comparable to those of mice inoculated with the RG *wt* virus, including lethargy, ruffled fur, and weight loss. Infection with H1N1pdm09-NLuc was associated with a slight delay in weight loss kinetics (Fig. 1D). However, the median lethal dose of H1N1pdm09-NLuc (10^4.5^ 50% tissue culture infective doses [TCID_50_]; Fig. 1E) was comparable to that of the RG *wt* virus and to the reported median lethal dose of the biological *wt* parental virus in BALB/c mice (10^4.7^ TCID_50_) (13). Furthermore, the infectious virus titers in the lung homogenates of mice inoculated with 10^3^ TCID_50_ of either H1N1pdm09-NLuc or H1N1pdm09 RG *wt* virus were comparable over the course of infection (Fig. 1F). Therefore, we concluded that the H1N1pdm09-NLuc virus is a suitable challenge virus for preclinical efficacy studies.

### Real-time dynamics of imaging in naive mice.

**FIG 2:**
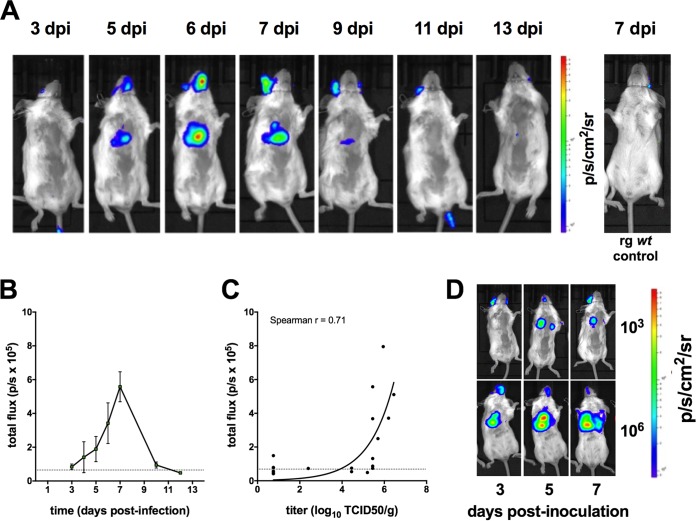
Bioluminescent *in vivo* imaging of H1N1pdm09-NLuc replication in naive mice. Adult BALB/c mice were inoculated intranasally with 10^3^ TCID_50_ of H1N1pmd09-NLuc virus. (A) A representative image series of a single mouse imaged at the time points indicated. A separate control mouse that was inoculated with 10^3^ TCID_50_ of H1N1pmd09 RG *wt* virus and imaged at 7 dpi is shown at the far right for comparison. (B) Bioluminescence kinetics (mean ± standard error of the mean) are shown for the course of sublethal infection (*n* = 4 mice) and expressed in photons per second per square centimeter per steradian (p/s/cm^2^/sr). (C) Cohorts of mice four per group) were imaged and then immediately euthanized at several time points. The infectious virus titer in the lungs was plotted against the bioluminescent signal for each animal. The time points represented are 3, 5, 7, 10, and 12 dpi. The dashed horizontal line indicates the limit of detection of the bioluminescent signal. (D) Mice were challenged with either a sublethal (10^3^ TCID_50_) or a lethal (10^6^ TCID_50_) inoculum of H1N1pdm09-NLuc virus and imaged at the time points indicated.

Naive BALB/c mice were inoculated intranasally with a sublethal dose (10^3^ TCID_50_) of H1N1pdm09-NLuc. Robust bioluminescence over the chest was detected in all inoculated mice. The background in the region of interest was negligible, although occasional background was observed at the luciferase substrate injection site. The full course of sublethal infection could be tracked by bioluminescence imaging (Fig. 2A). The peak signal level was reached in most animals at 7 dpi (Fig. 2B). In a separate experiment, cohorts of mice were imaged and necropsied at 3, 5, 7, 10, and 12 days postinfection (dpi) (four mice per time point) to correlate the bioluminescent signal from the chest with virus replication data. Infectious virus titer determination confirmed that an increased bioluminescent signal intensity corresponded to a high virus titer in lung tissue (Fig. 2C). Furthermore, we demonstrated that inoculation of a lethal dose (10^6^ TCID_50_) of virus resulted in earlier detection of bioluminescence in the chest and more profound bioluminescence than a sublethal dose (Fig. 2D) on the day of peak replication, illustrating the dynamic range of this system.

### Dynamics of bioluminescence in vaccinated mice.

**FIG 3:**
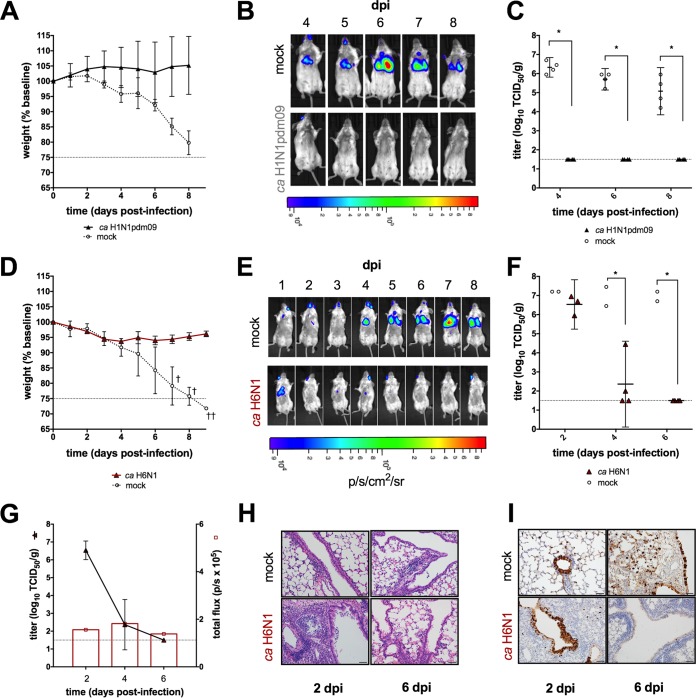
Bioluminescent *in vivo* imaging of vaccinated mice. Adult BALB/c mice were inoculated intranasally with a single dose of homologous or heterologous LAIV or mock vaccinated with L-15. All mice were challenged with 10^6^ TCID_50_ of H1N1pmd09-NLuc virus. Weight loss (A, D), a bioluminescence imaging series of a single representative mouse from each group (B, E), and virus titers in the lungs (C, F) are shown for homologous and heterosubtypic LAIV groups, respectively. The bioluminescent signal is expressed in photons per second per square centimeter per steradian (p/s/cm^2^/sr). (G) Cohorts of mice (*n* = 4 and *n* = 2 for the heterologous LAIV and mock-vaccinated groups, respectively) were imaged and then euthanized at 2, 4, and 6 dpi. Infectious virus titers (mean ± standard deviation) in the lung were plotted together with bioluminescent signal levels (mean flux in photons per second [p/s]). Hematoxylin and eosin staining (H) and immunostaining (I) of lung tissue sections collected at the times indicated were performed. Dashed lines indicate the threshold for euthanasia due to weight loss associated with infection (A, D) or the limit of detection of infectious virus (C, F, G). *, *P* < 0.05.

To determine whether IVIS could discriminate between the levels of protection conferred by matched and mismatched vaccines, BALB/c mice were immunized with a single dose of either homologous or heterosubtypic live attenuated influenza vaccine (LAIV) and subsequently challenged with a lethal dose of H1N1pdm09-NLuc. Immunization with homologous LAIV was fully protective, without illness or death in vaccinated mice, while mock-vaccinated mice rapidly lost weight (Fig. 3A). Consistent with these observations, chest bioluminescence remained below the limit of detection in all vaccinated mice. In contrast, signal was detectable in mock-vaccinated mice at the earliest imaging time point (Fig. 3B). Imaging results were supported by infectious virus titers in the lungs (Fig. 3C).

To determine whether IVIS could detect virus replication in the context of heterosubtypic vaccine-mediated protection, we imaged mice vaccinated with a cold-adapted (*ca*) H6N1 vaccine following a challenge with H1N1pdm09-NLuc. Cohorts of mice were euthanized at 2 and 4 dpi to determine the impact of heterosubtypic vaccine-mediated protection on the replication kinetics of the challenge virus early in the course of infection. Vaccinated mice experienced minor weight loss but did not succumb to a lethal challenge, in contrast to mock-vaccinated controls (Fig. 3D). In vaccinated mice, we observed a reduction in bioluminescence signal intensity compared to mock-vaccinated mice, which was statistically significant at 4 dpi (*P* = 0.007); in contrast, chest bioluminescence continued to increase in mock-immunized mice until 7 dpi (Fig. 3E). Consistent with observations from imaging, virus titers in the lungs of mice vaccinated with *ca* H6N1 LAIV were significantly lower than those in the lungs of mock-vaccinated mice at 4 dpi (*P* = 0.007). In fact, the challenge virus was not detected in two of four *ca* H6N1 LAIV-vaccinated mice at 4 dpi (Fig. 3F).

The bioluminescent signal kinetics were generally consistent with virus titer kinetics in mock-vaccinated mice. However, the absolute correlation between these two variables in mice that had been vaccinated with *ca* H6N1 LAIV was poor. In particular, chest bioluminescence was weak at early time points, despite the high virus titers observed in the lungs of these mice (Fig. 3G). Interestingly, histopathological analysis revealed evidence of severe pulmonary inflammation at 2 dpi, characterized by perivascular cuffing, alveolar inflammation and occasional necrotic debris (Fig. 3H). Furthermore, viral antigen staining revealed that prior vaccination with *ca* H6N1 LAIV was associated with reduced spread of the challenge virus from the airways into the parenchyma, in contrast to mock-vaccinated mice (Fig. 3I).

### Dynamics of bioluminescence in mice receiving antibody for prophylaxis or therapy.

Passive immunization is an attractive approach for the clinical management of severe disease associated with seasonal and pandemic influenza viruses. Several human MAbs (hMAbs) with potent neutralizing activity and broad cross-reactivity have been described recently (14), including an hMAb designated EM4CO4 that was isolated from an individual infected during the 2009 pandemic. This H1N1pdm09-specific hMAb was protective *in vivo* when administered prophylactically and also provided some benefit when administered therapeutically in mice (15). However, differences in viral clearance kinetics between mice receiving hMAb immunoprophylaxis versus immunotherapy have not been well described. To investigate whether bioluminescent imaging is sufficiently sensitive to discriminate between these two approaches, mice were given hMAb EM4CO4 either 24 h prior to or 72 h following a challenge with H1N1pdm09-NLuc.

**FIG 4:**
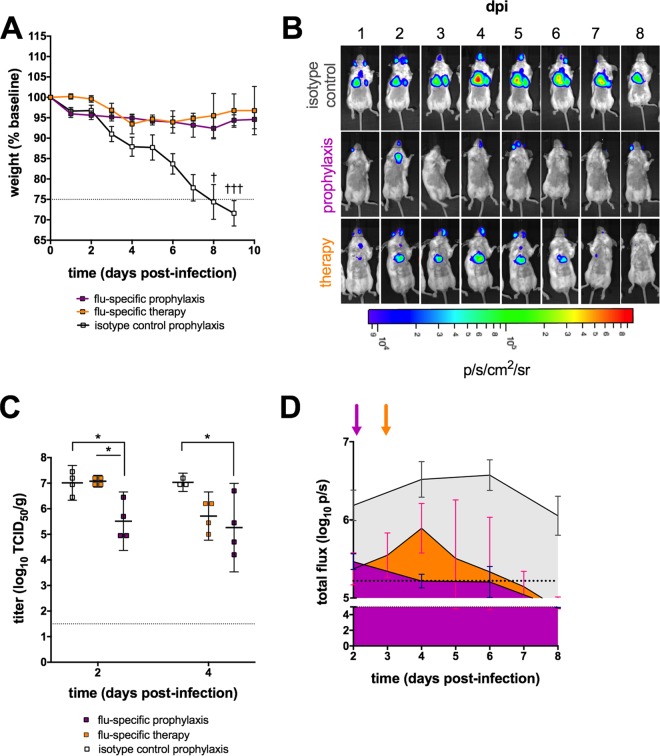
Bioluminescence *in vivo* imaging of passively immunized mice. An influenza virus-specific hMAb (EM4CO4) was administered either prophylactically (24 h prechallenge) or therapeutically (72 h postchallenge). Mice that received prophylaxis with an equivalent dose of isotype control antibody (human IgG1κ) are shown for comparison. (A to C) Weight loss (A); a bioluminescence imaging series of a single representative mouse from each group, expressed in photons per second per square centimeter per steradian (p/s/cm^2^/sr) (B); and virus titers in the lungs (C) are shown. (D) The mean bioluminescence in each group (four mice per group) is plotted for each time point indicated, and the area under the curve for each group is shaded to highlight differences in flux kinetics (photons per second). Magenta, prophylaxis; orange, therapy; gray, isotype control prophylaxis. Shaded arrows indicate the timing of antibody administration for each group that received influenza virus-specific antibody. Dashed lines indicate the threshold for euthanasia due to weight loss associated with infection (A), the limit of detection of infectious virus (C), or the limit of detection of the bioluminescent signal (D). *, *P* < 0.05.

Protection was observed in all passively immunized mice, regardless of the timing of hMAb administration (Fig. 4A). Prophylaxis did not completely prevent infection, as evidenced by detection of chest bioluminescence (Fig. 4B) and of virus in the lungs (Fig. 4C). Bioluminescence was significantly lower in mice that received hMAb prophylaxis than in isotype control mice by 4 dpi and remained lower thereafter (*P* = 0.04). IVIS was also able to reveal changes over the course of infection in the immunotherapy group. Chest bioluminescence was evident in this group beginning at 1 dpi and peaked at 4 dpi, 24 h following influenza virus hMAb administration. By 5 dpi, a statistically significant reduction in bioluminescence was evident in the hMAb-treated mice compared to that in mice that received the isotype control antibody (*P* = 0.03). Furthermore, IVIS could discriminate distinct patterns of bioluminescent signal kinetics among the three groups (Fig. 4D).

### Imaging infection in mice receiving stem versus head antibody prophylaxis.

The influenza virus HA is the major target of the human antibody response. Antibodies targeting conserved epitopes in the HA stem domain have been the subject of great interest in the context of universal influenza vaccine development (16–18). Recent evidence suggests that head- and stem-directed antibodies may neutralize influenza virus by distinct mechanisms (19–21). We investigated whether prophylaxis with an HA head-specific hMAb (EM4CO4) versus a stem-specific hMAb (70-1F02) would result in different kinetics of challenge virus clearance and whether IVIS can distinguish such differences. These antibodies were previously shown to be efficacious in mice, but their impact on the kinetics of virus replication has not been described.

**FIG 5:**
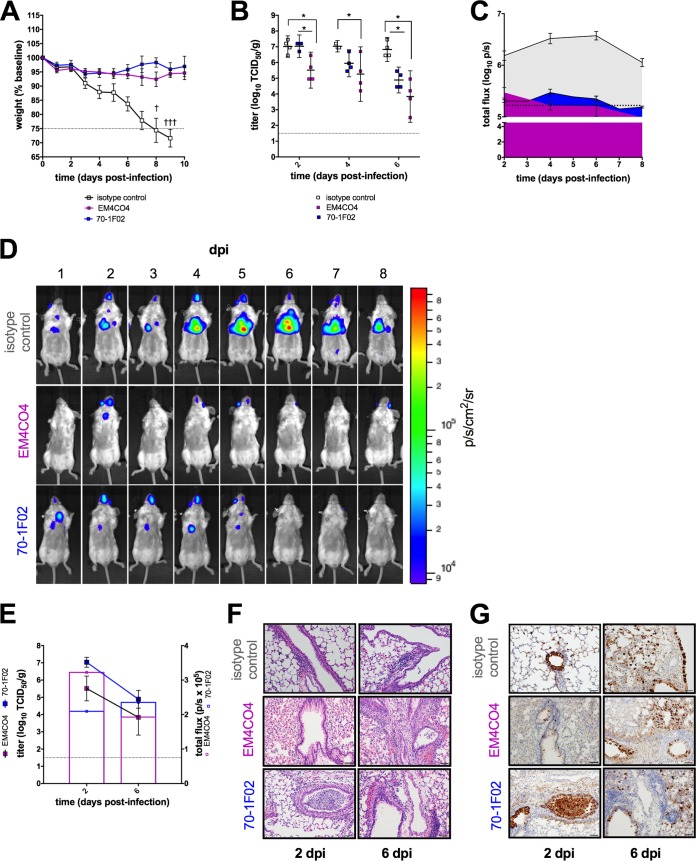
*In vivo* bioluminescence imaging of mice receiving passive immunotherapy with an HA head versus stem antibody. The levels of protection conferred by hMAbs, one directed to the HA head (EM4CO4), and one directed to the HA stem (70-1F02), were compared (isotype control, IgG1κ, included for comparison). (A to C) Weight loss (A), virus titers in the lungs (B), and kinetics of chest bioluminescence (C) are shown for the time points indicated (four per group) and are expressed in photons per second per square centimeter per steradian (p/s/cm^2^/sr). (D) A representative image series of a single mouse is shown for each group. (E) Cohorts of mice (four per group) were imaged and then immediately euthanized at 2, 4, and 6 dpi. Infectious virus titers in the lungs (mean ± standard deviation) were determined and plotted together with bioluminescent signal levels expressed in photons per second (p/s). (F, G) Hematoxylin and eosin staining (F) and immunostaining (G) of lung tissue sections collected at the time points indicated were performed. Dashed lines indicate the threshold for euthanasia due to weight loss associated with infection (A), the limit of detection of infectious virus (B, E), or the limit of detection of the bioluminescent signal (C). *, *P* < 0.05.

Prophylaxis with either hMAb was protective; passively immunized mice experienced little weight loss, in contrast to mice that received isotype control antibody (Fig. 5A). Infectious virus titers in the lungs of mice that received either influenza virus-specific hMAb were lower than the titers in recipients of isotype control antibody at 6 dpi (*P* < 0.05 for each), consistent with accelerated clearance of the challenge virus (Fig. 5B). Furthermore, lung titers of the challenge virus were statistically significantly lower as early as 2 dpi in the group of mice that received head-specific hMAb EM4CO4 than those in the group that received stem-specific hMAb 70-1F02 (*P* = 0.02).

Similarly, IVIS signal kinetics followed distinct patterns in the three groups (Fig. 5C). Chest bioluminescence persisted longer in mice that received prophylaxis with the stem antibody than in mice that received prophylaxis with the head antibody (Fig. 5D). Again, the correlation between the bioluminescence signal intensity and the infectious virus titer was not uniform over time in the head and stem antibody groups (Fig. 5E). Interestingly, a shift in viral antigen distribution was observed over the course of infection. At 2 dpi, viral antigen was predominantly associated with small and medium-size airways (bronchi). In contrast, at 6 dpi, virus antigen was located largely within the alveolar interstitium and was associated with a diminished inflammatory response (Fig. 5F and G). Immunoprophylaxis with either influenza virus-specific hMAb was associated with the presence of less antigen in the pulmonary parenchyma than in that of isotype control mice. These observations are consistent with the conclusions supported by imaging and infectious virus titration.

## DISCUSSION

Influenza virus infection can be associated with serious illness and death (2). Novel medical countermeasures are necessary in the face of the risk of antiviral resistance, serious complications associated with seasonal influenza virus infection, and the threat of pandemic influenza. Preclinical development of candidate vaccines and pharmaceuticals requires evaluation in relevant animal models (3), and new tools for such evaluation are important. We present evidence that noninvasive bioluminescent imaging can be used to track virus replication in real time in immunized mice, that it reliably predicts clinical and virologic outcomes following a challenge, and that it can discriminate between distinct patterns of protection conferred by different interventions.

IVIS had adequate sensitivity and dynamic range to track the dynamics of a full course of sublethal infection (Fig. 2A) and bioluminescence correlated with infectious virus titers in the lungs of immunologically naive mice (Fig. 2C). These observations illustrate the utility of our NLuc reporter virus, which is brighter, less attenuated, and more stable than other recently described luciferase reporter viruses constructed with the *Gaussia* luciferase (8, 22). Actively and passively immunized mice challenged with a lethal dose of bioluminescent virus could easily be distinguished from mock-vaccinated and isotype antibody-treated control mice by bioluminescence (Fig. 3B and E). Notably, IVIS can be informative even when weight loss kinetics are not, as we observed when comparing passive immunotherapy with head- and stem-specific antibodies (Fig. 5A and D). Changes in bioluminescence preceded changes in weight loss and virus titer, demonstrating the utility of this system for predicting clinical and virologic outcomes. IVIS thus enhances the assessment provided by traditional study methods. Furthermore, IVIS offers opportunities to track virus replication in several organs simultaneously and this may provide insight into mechanisms of protection. For example, inspection of IVIS images suggests that replication of the challenge virus in the upper respiratory tract was controlled better by LAIV than by administration of either MAb; this likely reflects mucosal immunity induced by LAIV.

However, we propose several important considerations for the design of efficacy studies based on *in vivo* imaging technology. First, the specific design of the reporter construct, the gene segment into which it was inserted, the subtype of the virus, and how the recombinant virus was rescued and grown may all influence attenuation, virulence, and genetic stability. Several reporter influenza A viruses have been described in recent years and have been found to have distinct properties, particularly with respect to genetic stability and attenuation (5, 7, 10, 12, 23, 24). The replication and virulence of these reporter viruses must therefore be carefully evaluated *in vivo*, particularly when a study design includes several challenge viruses.

Second, the utility of IVIS is critically influenced by the fidelity of bioluminescent signal transmission. As we have described in this report, the peak signal of the reporter virus may not coincide with peak replication measured by titration of infectious virus. This may be because of properties inherent to the reporter virus system that have yet to be defined or may reflect the effect of host factors. Although luciferase is an attractive reporter for *in vivo* imaging applications because of the inherently low background level in mammalian tissue, many common luciferases emit blue-shifted light (25). Hemoglobin strongly absorbs visible light in the green and blue regions of the spectrum and is therefore a major contributor to signal attenuation (26). Fur also modifies signal via scattering of emitted light (27). Finally, the ability to track virus replication in the lower respiratory tract is particularly important for efficacy studies because severe influenza illness is often associated with lower respiratory tract involvement (28, 29). However, overlying tissue substantially attenuates light transmission, as demonstrated by *ex vivo* or *in situ* imaging experiments with reporter viruses and isotropic light-emitting beads (6, 25, 27, 30). The NLuc luciferase used in our reporter virus is the brightest commercially available luciferase, permitting detection of robust signal despite these limitations (4, 31). Nevertheless, each of these factors imposes constraints on the dynamic range of IVIS signal relative to the dynamic range of virus titration.

Third, we found that the correlation between chest bioluminescence and infectious virus titers quantified from the lungs was variable among study groups. For example, bioluminescence in mice vaccinated with *ca* H6N1 LAIV was lower at early time points than we would have predicted on the basis of the observed virus titers, leading to a poor correlation between lung infectious titers and chest bioluminescence (Fig. 3). Interestingly, histopathological analysis of the lungs revealed enhanced inflammation at 2 dpi. This is consistent with previous observations that heterosubtypic protection elicited by vaccination of mice with LAIV is mediated by rapid recruitment of cytotoxic T cells (32). The impact of inflammatory infiltrate on bioluminescent signal attenuation *in vivo* is not well understood, but increased circulation and vascular leakage are hallmarks of inflammation and edema has previously been reported to attenuate measurable signals (25). Finally, viral antigen distribution shifts over the course of infection from predominantly peribronchial to disseminated throughout the parenchyma in immunologically naïve, as well as immunized, mice. It is unclear whether the dynamics of antigen distribution throughout the lungs may influence the fidelity of bioluminescent signal quantification, but the possibility of such confounding factors should be considered when designing IVIS studies.

In summary, we demonstrate that IVIS enhances the information obtained by traditional methods of viral load measurement in a mouse model of influenza. To our knowledge, this is the first demonstration of the application of IVIS technology in the context of longitudinal preclinical efficacy studies for influenza. IVIS has many potential applications as a means to track challenge virus replication in mice. The ability to observe mice longitudinally makes it an especially powerful tool for identifying evidence of protection with interventions that may not immediately affect virus titer or weight, e.g., in the context of heterosubtypic protection or postexposure prophylaxis. Furthermore, the ability of IVIS to discern distinct kinetics of challenge virus replication associated with different interventions illustrates its potential as a method to support mechanistic studies or to facilitate early detection of the emergence of antiviral resistance or immune escape while reducing the number of mice necessary for such analyses. Although imaging data should be supported by virus titration, tracking of virus replication in immunized mice by *in vivo* imaging holds great promise as a complementary strategy to enhance conventional preclinical efficacy studies.

## MATERIALS AND METHODS

### Plasmids and cells.

The eight-plasmid RG system for A/California/07/2009 (A/CA/07/09) and the reporter construct have been described previously (7, 33). Briefly, the reporter construct was generated by introduction of the coding sequence for the luciferase NLuc downstream of the PA polymerase subunit sequence, which has been shown to tolerate the C-terminal insertion of short exogenous gene sequences (7, 11, 34, 35). A 2A cleavage site and other sequence modifications were also introduced into this construct, designated PASTN, to ensure stable NLuc expression (7). The stability of luciferase-expressing reporter viruses generated with the PASTN construct has been previously demonstrated (6, 7). 293T and MDCK cells were maintained in Dulbecco’s modified Eagle’s medium (Gibco, Grand Island, NY) and Eagle’s minimum essential medium (Lonza, Walkersville, MD), respectively, supplemented with 40 mM l-glutamine (Gibco, Grand Island, NY) and 10% heat-inactivated fetal bovine serum (HyClone, Logan, UT). All cells were cultured at 37°C in a humidified atmosphere with 5% CO_2_.

### Generation of reporter virus.

To produce the RG A/CA/07/09 reporter virus (H1N1pdm09-NLuc), a 293T-MDCK cell coculture was transfected with 1 μg of each plasmid encoding the seven gene segments of A/CA/07/09 and the PA-NLuc reporter construct by the lipofection method with TransIT-LT1 (Mirus Bio LLC, Madison, WI) in Opti-MEM (Gibco, Grand Island, NY). Supernatant was collected at 72 h posttransfection, cleared by centrifugation, tested for hemagglutination activity with turkey red blood cells (Lampire, Everett, PA), and used to inoculate eggs (see below). As a control, RG *wt* A/CA/07/09 was generated with the same protocol.

### Reporter virus propagation and titration.

Stock virus was prepared via propagation for two passages in the allantoic cavities of 10- to 11-day-old embryonated chicken eggs (Charles River, Inc. SPAFAS, Franklin, CT). Allantoic fluid was harvested at 72 h postinoculation, cleared by centrifugation, and stored in aliquots at −80ºC until used. The TCID_50_ was determined by inoculation of serial 10-fold dilutions of stock virus onto MDCK cells as described previously (36), and the titer was calculated by the Reed-Muench method (37). Multicycle replication assays were performed by inoculation of MDCK cells in triplicate with either RG *wt* H1N1pdm09 or H1N1pdm09-NLuc virus at a multiplicity of infection (MOI) of either 0.01 or 0.001, and the medium was supplemented with 0.1% bovine serum albumin (Sigma, St. Louis, MO) and bovine pancreatic trypsin treated with l-(tosylamido-2-phenyl) ethyl chloromethyl ketone (TPCK; Worthington Biochemical Corp., Lakewood, NJ). Supernatant was sampled every 24 h until 72 h postinoculation. The virus titer in the supernatant was determined on fresh MDCK cells. Luminescence was measured on a SpectraMax M5 plate reader (Molecular Devices, Sunnyvale, CA) after the addition of nano-Glo substrate (Promega, Madison, WI) in accordance with the manufacturer’s directions. The threshold of detection was calculated as the mean plus three standard deviations of the luminescence from the supernatant of mock-inoculated wells and is indicated with a dashed line.

### Vaccines and antibodies.

The *ca* A/teal/HK/W312/97 (H6N1) and *ca* A/CA/07/09 (H1N1pdm09) viruses have been previously described (38, 39). Stock viruses were passaged once in 10- to 11-day-old embryonated hen eggs, and aliquots were stored at −80°C until used. hMAbs EM4CO4 and 70-1F02 were isolated from individuals infected with the 2009 H1N1 pandemic virus and were provided by R. Ahmed of Emory University. These MAbs have been previously described (15). Human IgG1κ (Sigma, St. Louis, MO) was utilized as an isotype control antibody and administered at an identical dose in an identical manner.

### Animal studies.

All of the animal experiments described below were conducted under protocols approved by the IACUC at the National Institute of Allergy and Infectious Diseases, NIH.

### Virulence.

To assess the relative virulence of H1N1pdm09-NLuc, 6- to 8-week-old female BALB/c mice (Taconic Biosciences, Albany, NY) were lightly anesthetized with isoflurane and inoculated intranasally with 50 μl of serial 10-fold dilutions of 10^1^ to 10^6^ TCID_50_ of H1N1pmd09-NLuc or 10^4^ to 10^6^ TCID_50_ of RG *wt* H1N1pdm09 (five mice per group). Mice were monitored daily for 14 days to record clinical signs and weights. Mice were humanely euthanized if they lost 25% or more of their initial body weight (the weight loss threshold is indicated in relevant figures by a dashed line, and each cross symbol denotes euthanasia of a single mouse at the time point indicated). The median lethal dose of each virus was calculated as previously described (13).

### Active and passive immunizations.

Four- to 6-week-old female BALB/c mice (Taconic) were lightly anesthetized with isoflurane and inoculated intranasally with 10^6^ TCID_50_ of either *ca* A/CA/07/09 (H1N1pdm09) or *ca* A/teal/HK/W312/97 (H6N1) LAIV in 50 μl in groups of 12 or 16 mice as indicated. An equivalent volume of Leibovitz’s L-15 medium (Lonza, Walkersville, MD) was administered intranasally to mock-immunized mice. Mice were challenged 4 weeks later by intranasal inoculation of 10^6^ TCID_50_ of H1N1pdm09-NLuc in 50 μl. Alternatively, mice were injected intraperitoneally with hMAb at 10 mg/kg diluted in phosphate-buffered saline (PBS) either 24 h prior to a virus challenge or 72 h postchallenge. Weights were monitored daily, and mice were humanely euthanized if they lost 25% or more of their initial body weight. Mice were imaged daily until necropsy or until 10 days postchallenge, as indicated. Groups of four mice were humanely euthanized 2, 4, or 6 days postchallenge; lungs were collected for virus titration and homogenized; and virus titers were determined as described previously (38). The limit of detection of the infectious virus titer or bioluminescent signal is indicated in relevant figures by a dashed line.

### *In vivo* imaging.

Bioluminescent imaging was performed with an IVIS 100 (Xenogen, Alameda, CA). Mice were lightly anesthetized with isoflurane. Nano-Glo luciferase substrate (Promega, Madison, WI) was diluted 1:20 in PBS and injected retro-orbitally in a volume of 100 μl. Mice were immediately imaged for 5 min. Isoflurane anesthesia was maintained over the course of imaging. Bioluminescence data were acquired and analyzed with Living Image software (PerkinElmer, Waltham, MA). Images are uniformly scaled. The threshold of detection of luminescence was calculated as the mean plus 3 standard deviations of the region of interest over the thorax imaged at 12 dpi.

### Pathology and immunohistochemistry.

Lungs were fixed in 10% buffered formalin (VWR, Radnor, PA) and embedded in paraffin. For pathology, serial sections (5 µm) were stained with hematoxylin and eosin. For immunohistochemistry, staining was carried out on the Bond RX (Leica Biosystems, Buffalo Grove, IL) platform in accordance with a modified manufacturer-supplied protocol. Briefly, sections were deparaffinized and rehydrated. Heat-induced epitope retrieval was performed with epitope retrieval solution 2 (pH 10), and slides were heated to 100°C for 20 min. The specimen was then incubated with hydrogen peroxide to quench endogenous peroxidase activity; this was followed by application of the primary antibody, rabbit anti-influenza virus nucleoprotein (GeneTex, Irvine, CA) at a 1:4,000 dilution for 15 min at room temperature. Detection with horseradish peroxidase-conjugated anti-rabbit antibody, 3,3'-diaminobenzidine chromogen, and counterstaining with hematoxylin was completed with the Bond Polymer Refine Detection kit (Leica Biosystems). Slides were cleared through gradient alcohol and xylene washes prior to mounting and coverslipping. Sections were examined by light microscopy with an Olympus BX51 microscope, and photomicrographs were taken with an Olympus DP73 camera. Slides were reviewed by a veterinary pathologist who was unaware of the study groups. Images are shown at ×20 magnification.

### Statistical analysis.

Summary data shown represent the mean ± the 95% confidence interval (CI), unless otherwise indicated. Comparison of groups with regard to virus titers and bioluminescent flux values (photons per second) was performed by one-way analysis of variance and Welch’s *t* test with Holm-Sidak correction for multiple comparisons. Analysis of the correlation between virus titers and flux was performed by calculation of Spearman’s rank correlation coefficient, and their relationship was modeled by nonlinear regression. All analysis was performed in Prism v 7 (GraphPad, La Jolla, CA). A comparison was considered to be statistically significant when the *P* value was <0.05, as is indicated by an asterisk.

10.1128/mBio.00714-17.1 FIG S1  Weight loss kinetics in immunologically naive mice. (A, B) BALB/c mice (five per group) were inoculated intranasally with 10^4^, 10^5^, or 10^6^ TCID_50_ of H1N1pdm09 RG *wt* virus (A) or 10^1^ to 10^6^ TCID_50_ of H1N1pdm09-NLuc virus (B). Daily weight loss (mean ± standard deviation) relative to the baseline is indicated for each time point. Dashed lines indicate the threshold for euthanasia due to weight loss associated with influenza virus infection. Download FIG S1, TIF file, 1.2 MB. Copyright © 2017 Czakó et al. 2017 Czakó et al. This content is distributed under the terms of the Creative Commons Attribution 4.0 International license.
