# Supplementary figures and images for: In Vivo Imaging of Influenza Virus Infection in Immunized Mice
Source: mBio. 2017 May 30;8(3):e00714-17. doi: 10.1128/mBio.00714-17 (PMC5449660; doi:10.1128/mBio.00714-17)

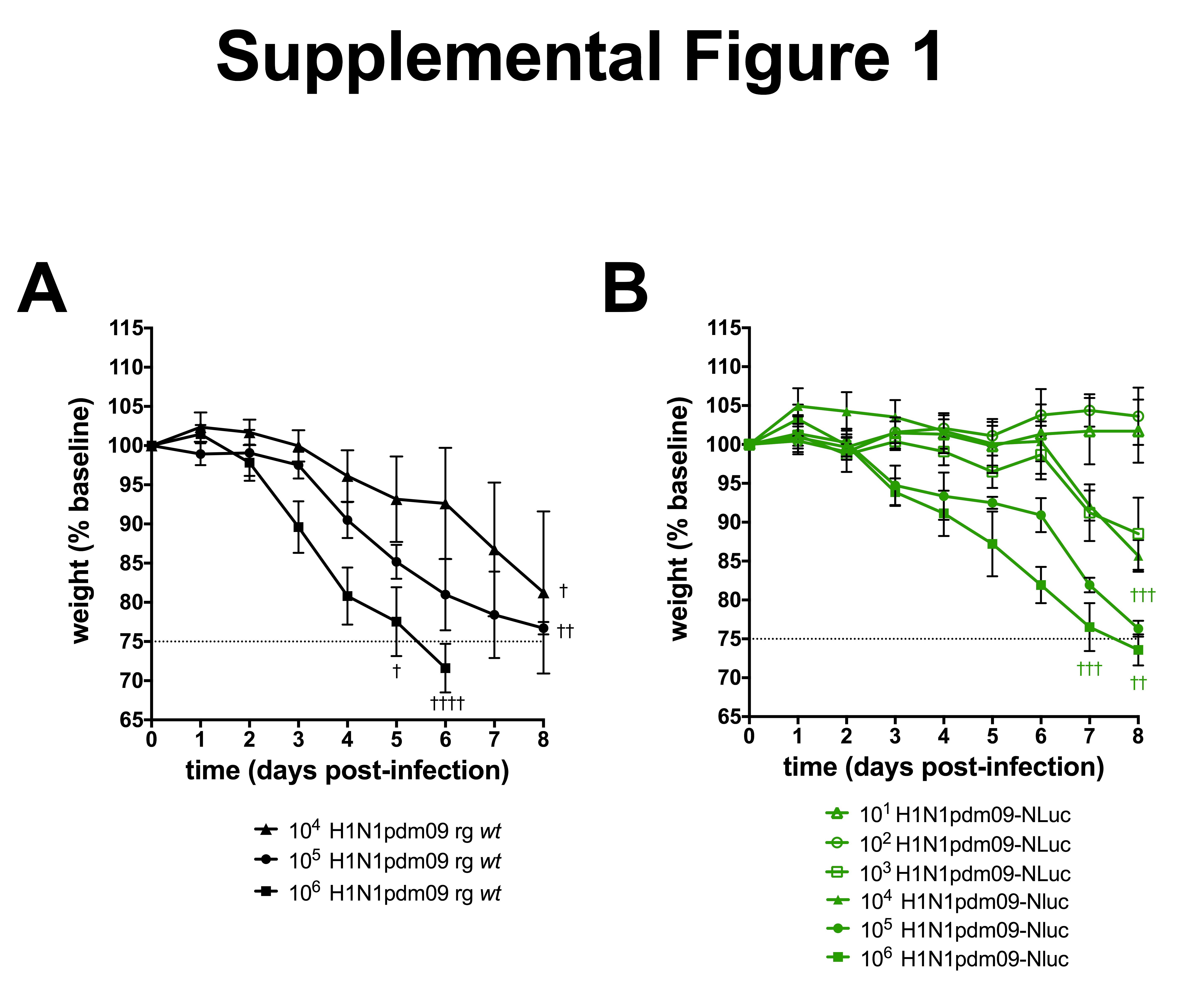

Supplement: FIG S1 [file mbo003173321sf1.tif]
